# Predictive value of lymphocyte-associated inflammation index in post-stroke cognitive impairment: a systematic review and meta-analysis

**DOI:** 10.3389/fneur.2024.1469152

**Published:** 2025-01-15

**Authors:** Feng-le Mao, Xia He, Xia-lian Huang, Yue-ming Cheng, Fu-li Qin, Yan-qiu Wang

**Affiliations:** ^1^School of Health Preservation and Rehabilitation, Chengdu University of Traditional Chinese Medicine, Chengdu, China; ^2^Affiliated Sichuan Provincial Rehabilitation Hospital of Chengdu University of Traditional Chinese Medicine, Chengdu, China

**Keywords:** post-stroke cognitive impairment, lymphocyte, inflammation index, systematic review, meta-analysis

## Abstract

**Background:**

The predictive role of the lymphocyte-associated inflammation index in post-stroke cognitive impairment (PSCI) remains controversial. Therefore, we performed an updated meta-analysis to update the evidence.

**Methods:**

This meta-analysis was conducted following the Preferred Reporting Items for Systematic Reviews and Meta-Analyses (PRISMA) guidelines. Six databases were systematically searched from their inception to May 5, 2024. Two investigators independently conducted literature screening and data extraction for the included studies. Two investigators independently assessed the quality of the included studies using the Newcastle-Ottawa Scale (NOS). Combined effect sizes were calculated using weighted mean difference (WMD) or standardized mean difference (SMD) with 95% confidence intervals (CIs). Heterogeneity was tested using the chi-square (χ2) test (Cochran’s Q) and index of inconsistency (*I*^2^), Publication bias was assessed using funnel plots and Egger’s regression test.

**Results:**

This systematic review included a total of 16 studies, encompassing 3,406 patients. Meta-analysis revealed that neutrophil-to-lymphocyte ratio (NLR) levels were significantly higher in the PSCI group compared to the non-PSCI group (WMD: 1.12; 95% CI: 0.85, 1.40; *p* < 0.00001). Similarly, the platelet-to-lymphocyte ratio (PLR) levels were significantly higher in the PSCI group compared to the non-PSCI group (WMD: 16.80; 95% CI: 4.30, 29.29; *p* = 0.008). However, there was no statistically significant difference between the two groups concerning hemoglobin, albumin, lymphocyte, and platelet (HALP) scores (WMD: -12.78; 95% CI: −25.95, 0.38; *p* = 0.06) and lymphocyte count (WMD: -0.13; 95% CI: −0.34, 0.07; *p* = 0.20).

**Conclusion:**

Increased levels of PLR and NLR are strongly associated with the PSCI, which may serve as an effective tool for predicting PSCI. However, there is insufficient evidence to support a direct relationship between HALP scores, lymphocyte count, and PSCI.

**Systematic review registration:**

https://www.crd.york.ac.uk/prospero/, identifier CRD42023462232.

## 1 Introduction

Stroke, a prevalent cerebrovascular disease, has emerged as one of the foremost causes of disability and death worldwide, posing significant challenges to public health (1, 2). Post-stroke cognitive impairment (PSCI) is a clinical syndrome characterized by cognitive deficits following a stroke, with approximately one-third of stroke patients experiencing varying degrees of PSCI (3). Patients with PSCI have cognitive dysfunction, which leads to compromised motor, language, and self-care abilities, which in turn increases the family’s healthcare costs and severely impacts the patient’s quality of life (4). Studies have shown that the period from post-stroke to the onset of PSCI can be considered a therapeutic window for early intervention to protect cognitive function and reduce mortality through better early care (5, 6). Therefore, identifying validated predictors for early screening and evaluation of patients with PSCI is particularly crucial.

Inflammatory factors have a strong association with cognitive impairment (7, 8). Recent studies indicate that lymphocytes play a pivotal role in inflammatory repair and brain protection (9). Lymphopenia after stroke also suggests a poor prognosis for neurological disorders (10). Clinically, lymphocyte-associated inflammation indices such as the neutrophil-to-lymphocyte ratio (NLR), platelet-to-lymphocyte ratio (PLR), and HALP (hemoglobin, albumin, lymphocyte, and platelet) scores are strongly correlated with cardiovascular disease and malignancy (11, 12). However, it remains unclear whether these inflammatory markers are associated with cognitive impairment following stroke. Therefore, our study aimed to systematically retrieve and analyze studies on the lymphocyte-associated inflammation index to provide an evidence-based basis for its clinical application in the early prediction of PSCI.

## 2 Methods

This meta-analysis adheres to the PRISMA (Preferred Reporting Items for Systematic Reviews and Meta-Analyses) 2020 statement (13) and was registered prospectively in PROSPERO (CRD42024541099). The PRISMA 2020 checklist is presented in Supplementary Table 1.

### 2.1 Literature search

We systematically searched PubMed, Embase, the Cochrane Library, Web of Science, Chinese National Knowledge Infrastructure (CNKI), and Wanfang Database from their inceptions to May 5, 2024. We focused on studies related to the use of the lymphocyte-associated inflammation index to predict PSCI. The search terms included: “stroke,” “post-stroke cognitive impairment,” “lymphocytes,” “cognitive impairment,” and related terms. In addition, we manually screened the unpublished literature for data that might have met the inclusion criteria, including data from conferences, Preprint, and other sources, thus ensuring that all data that met the criteria were included. The detailed search strategy is presented in Supplementary Table 2. Two investigators (FLM and YMC) independently searched the reference lists of all identified articles and gray literature for potentially eligible studies.

### 2.2 Identification of eligible studies

Studies meeting the following criteria were included: (1) evaluations of the relationship between the lymphocyte-associated inflammation index and PSCI; (2) subjects were patients with or without cognitive impairment post-stroke; (3) observational study designs, including cohort and case–control studies; and (4) sufficient data on lymphocyte-associated inflammation index available for extraction. Studies meeting the following exclusion criteria were excluded: (1) duplicate publications, reviews, meta-analyses, and animal experiments; (2) studies with unavailable full texts or data; and (3) literature in languages other than English and Chinese.

### 2.3 Data extraction

Two investigators (FLM and YMC) independently extracted data from all eligible studies, disagreements were settled through consultation with an experienced investigator (HX). The collected data included the first author, year, study duration, region, study design, sample size, age, gender, PLR, NLR, HALP scores, and lymphocyte count. When continuous variables were reported as median with range or interquartile range, we used the validated mathematical method to calculate the mean ± standard deviation (14, 15). When data were missing or not reported in the study, we contacted the corresponding authors to obtain completed data if available.

### 2.4 Quality assessment

Two researchers (FLM and FLQ) independently assessed the quality of included studies using the Newcastle-Ottawa Scale (NOS) (16). The NOS includes three domains: selection, comparison, and exposure/outcome evaluation. The scale comprises 8 items and is scored out of 9, with a score of 6 or higher indicating a high-quality study. In case of disagreements, a third investigator (XH) was involved.

### 2.5 Statistical analysis

Statistical analysis for this study was conducted using Review Manager (version 5.4). As the lymphocyte-associated inflammation indices were continuous variables, standardized mean difference (SMD) or weighted mean difference (WMD) with 95% confidence intervals (CIs) were used as combined effect sizes. Heterogeneity was assessed using the chi-square (χ2) test (Cochran’s Q) and the index of inconsistency (*I*^2^) (17). If *p* > 0.05 or *I*^2^ ≤ 50%, the possibility of inter-study heterogeneity was considered small and meta-analysis was performed using a fixed-effects model; if *p* < 0.05 or *I*^2^ > 50%, the possibility of inter-study heterogeneity was considered large and meta-analysis was performed using a random-effects model. Forest plots were used to display the pooled estimates, and *p* < 0.05 was regarded as statistically significant.

### 2.6 Subgroup analysis

Subgroup analysis was conducted based on study design and country.

### 2.7 Sensitivity analysis

The present study used leave-one-out analysis to assess the effect of the included studies on the pooled results for outcomes with significant heterogeneity.

### 2.8 Publication bias

Egger regression test using Stata (version 12.0) and funnel plots using Review Manager (version 5.4) were used to assess publication bias when 10 or more studies were included (18).

## 3 Result

### 3.1 Literature search and study characteristics

**Figure 1:**
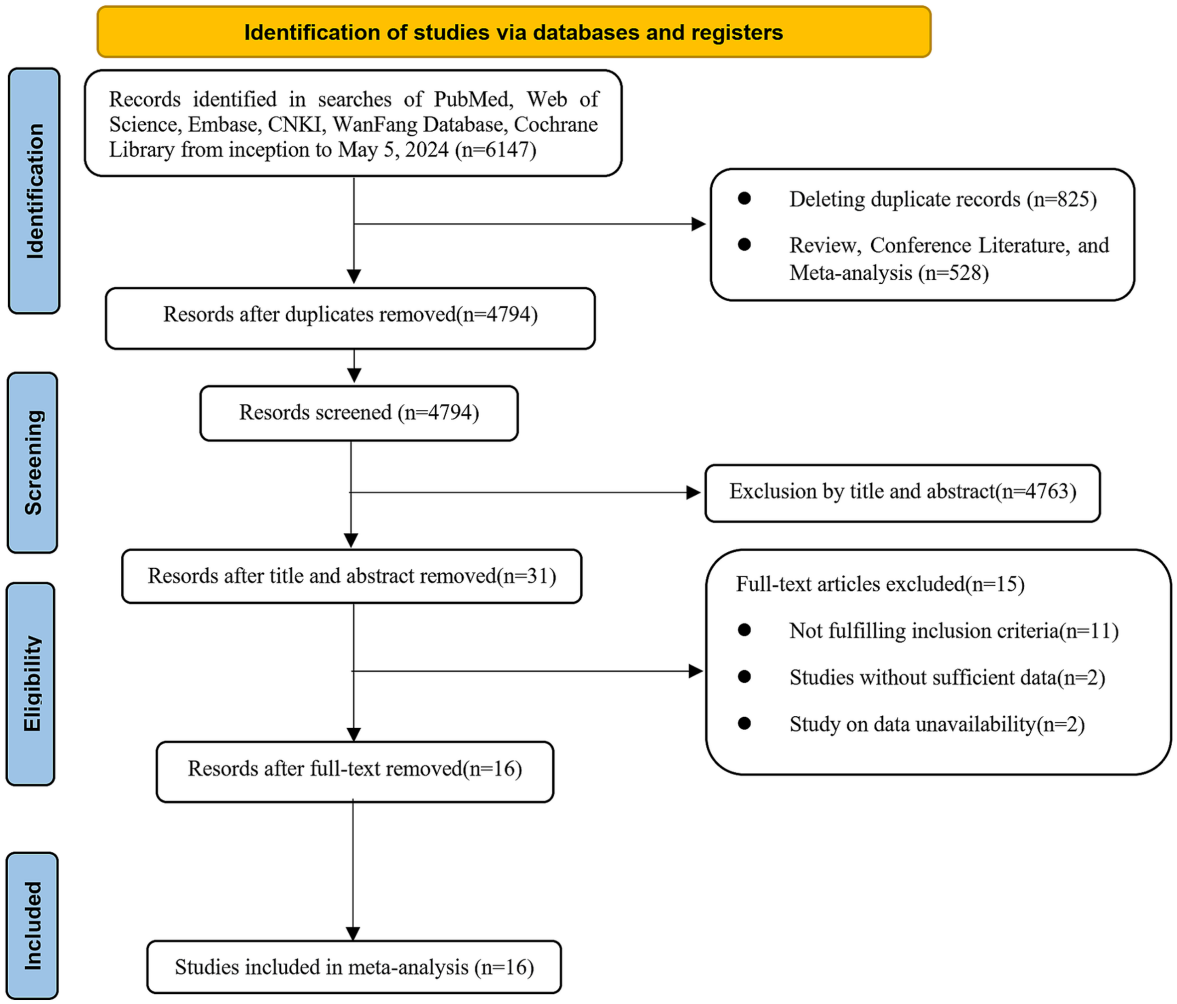
PRISMA flow chart of literature searching and screening.

We retrieved a total of 6,147 kinds of literature after conducting a systematic search. After excluding 825 duplicates and then performing an initial screening of titles and abstracts, 31 articles were identified as potentially relevant for this study. After full-text review and data extraction, 16 articles (19–34) including 3,406 patients were included in this study. Figure 1 illustrates the flowchart of the systematic retrieval and screening process. Table 1 summarizes the main characteristics of the included studies. Eight studies (22, 23, 25, 28–30, 33, 34) were cohort studies and eight were case–control studies (19–21, 24, 26, 27, 31, 32). The publications were primarily from 2020 to 2023, and the study populations mainly consisted of individuals aged 60–70 years. A total of 25 comparative groups were extracted from the 16 included papers because some of the included papers reported multiple comparative studies at the same time. Eleven studies compared NLR, six studies compared PLR, two studies compared HALP scores, and six studies compared lymphocyte counts. The median Newcastle-Ottawa Scale score for the 16 studies was 8 (range: 6–9, Table 2), with a range of 6–9 quality scores for the cohort studies and 8–9 for the case–control studies. Therefore, all included studies were considered to be of high quality and there were no low-quality studies.

**Table 1:** Characterization of the studies included in the systematic review.

Author/year	Study period	region	Patients with PSCI						
			Age	Male	Sample	NLR	PLR	HALP scores	Lymphocyte count
Fei Zha 2021	2012–2017	China	67.25 ± 8.80	37/50	87	2.73 ± 1.26			
Hongxv Wang 2023	2020–2022	China	NA	NA	100		162.9 ± 21.5		
Ke Dai 2023	2021–2022	China	68(63, 75)*	32/11	43				1.61 ± 0.55
Le Yang 2024	2019–2023	China	60–75	77/50	127	4.35 ± 1.02	132.81 ± 30.59		
Mingming Gao 2022	2019–2021	China	64.3 ± 4.2	22/16	38	6.4 ± 2.1			
Minjie Xu 2023	2017–2021	China	68.0(60.8, 74.0)*	153/229	382		126.8(103.6, 169.0)*	38.7(28.6, 52.1)*	
Minwoo Lee 2021	2010–2015	South Korea	66.7 ± 11.2	38/33	71	3.9 ± 3.0			
Shouwen Zhang 2024	2020–2023	China	66.7 ± 7.2	18/15	33	3.9 ± 0.6			
Tao Zhou 2024	2023	China	64.1 ± 12.3	69/37	106		148.35(117.12, 182.09)*	33.35 (27.30, 49.93)*	1.72(1.36, 2.02)*
Wenjun Ning 2023	2019–2022	China	65.88 ± 6.25	32/22	54	7.18 ± 2.47	3.52 ± 1.13		
Xiaomin Guo 2024	2021–2022	China	66.55 ± 1.37	20/9	29	2.42 ± 1.37			3.47 ± 1.50
Xiaoxing Li 2023	2020–2022	China	64.32 ± 9.82	40/21	61	6.9(4.1, 11.7)*			1.8(1.4, 2.2)*
Yanhong Xin 2023	2020–2022	China	62.99 ± 7.138	57/44	101	3.74(3.41, 5.15)*	131.78(122.63, 149.56)*		
Yanzhao Xie 2023	2021–2022	China	69(65, 73)*	46/31	77	2.93(2.29, 4.35)*			
Yongchun Wang 2024	2019–2022	China	63.0(58, 70)*	77/44	121			1.67 (1.31, 2.13)*	
Yuyan Wu 2023	2021–2022	China	64.38 ± 6.34	18/16	34	3.92 ± 1.51		2.01 ± 0.68	

Author/year	Non-PSCI patients						
	Age	Male	Sample	NLR	PLR	HALP scores	Lymphocyte count
Fei Zha 2021	60.12 ± 10.25	188/92	280	2.14 ± 0.80			
Hongxv Wang 2023	NA	NA	82		132.2 ± 16.8		
Ke Dai 2023	66.5(51, 74)*	32/6	38				1.59 ± 0.54
Le Yang 2024	60–75	106/93	199	3.43 ± 0.84	127.59 ± 28.30		
Mingming Gao 2022	56.8 ± 6.5	40/22	62	3.6 ± 1.0			
Minjie Xu 2023	63.0 (54, 69)*	61/139	210		121.9 (93.7, 150.8)*	45.4 (33.4, 59.2)*	
Minwoo Lee 2021	62.0 ± 12.0	184/90	274	2.7 ± 1.7			
Shouwen Zhang 2024	62.0 ± 6.8	92/45	137	2.7 ± 0.4			
Tao Zhou 2024	57.7 ± 11.1	91/23	114		113.83(94.81, 139.05)*	52.62 (41.01, 64.35)*	2.08 (1.66, 2.59)*
Wenjun Ning 2023	65.81 ± 6.94	16/10	26	5.41 ± 1.33	2.73 ± 0.74		
Xiaomin Guo 2024	63.91 ± 1.41	21/20	32	2.11 ± 0.65			3.61 ± 1.44
Xiaoxing Li 2023	60.55 ± 11.03	41/21	53	1.8(1.4, 2.2)*			1.5(1.2, 2.1)*
Yanhong Xin 2023	60.50 ± 8.605	93/60	153	3.15(2.80, 3.82)*	114.70(91.44, 134.52)*		
Yanzhao Xie 2023	63(57, 70)*	54/30	84	2.26(1.74, 2.64)*			
Yongchun Wang 2024	61.0 (55, 67)*	124/40	164				1.86 (1.48, 2.34)*
Yuyan Wu 2023	65.33 ± 6.19	20/14	34	2.81 ± 0.90			2.36 ± 0.72

^*^Median[range].

NA: not applicable.

PSCI, Post-stroke cognitive impairment; Non-PSCI, Non-post-stroke cognitive impairment; NLR, neutrophil-to-lymphocyte ratio; PLR, platelet-to-lymphocyte ratio; HALP, hemoglobin, albumin, lymphocyte, and platelet.

**Table 2:** Risk of bias assessment according to the Newcastle-Ottawa Scale.

Reference	Study design	Selection	Comparability	Exposure/Outcome	Total
Fei Zha 2021	Prospective cohort	****	*	***	8
Hongxv Wang 2023	Case–control	****	*	***	8
Ke Dai 2023	Case–control	***	**	***	8
Le Yang 2024	Case–control	****	*	***	8
Mingming Gao 2022	Prospective cohort	****	*	**	7
Minjie Xu 2023	Retrospective cohort	***	*	**	6
Minwoo Lee 2021	Retrospective cohort	***	*	***	7
Shouwen Zhang 2024	Prospective cohort	****	*	***	8
Tao Zhou 2024	Case–control	****	**	***	9
Wenjun Ning 2023	Case–control	****	*	***	8
Xiaomin Guo 2024	Case–control	***	*	***	7
Xiaoxing Li 2023	Prospective cohort	****	*	***	8
Yanhong Xin 2023	Prospective cohort	****	*	***	8
Yanzhao Xie 2023	Case–control	***	*	***	7
Yongchun Wang 2024	Prospective cohort	****	*	***	8
Yuyan Wu 2023	Case–control	****	**	***	9

****4 points; ***3 points; **2 points; *1 point.

### 3.2 The results of meta-analysis

#### 3.2.1 Correlation between NLR levels and PSCI

**Figure 2:**
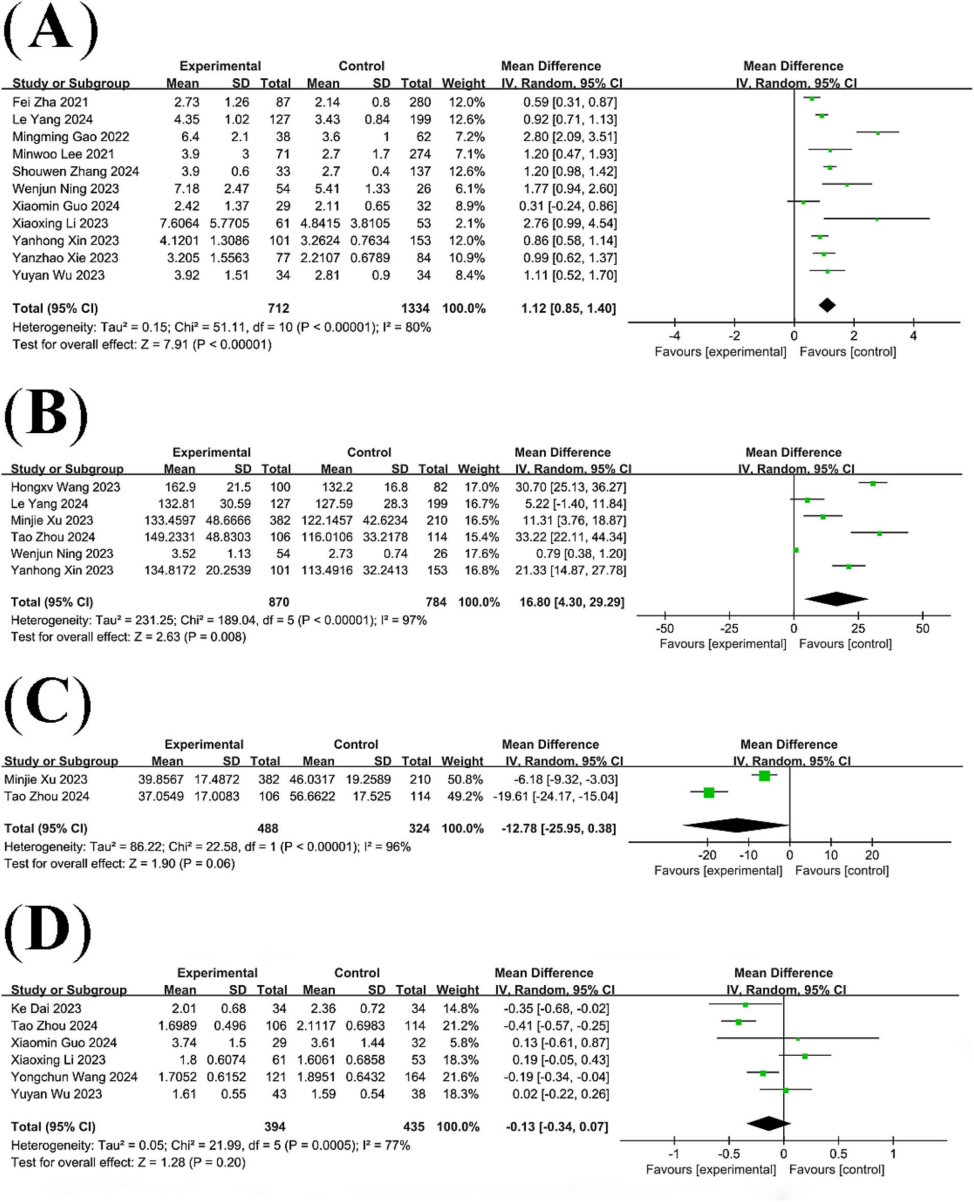
Forest plots of **(A)** NLR, **(B)** PLR, **(C)** HALP scores, and **(D)** lymphocyte count.

A total of 13 studies compared NLR levels in patients with post-stroke cognitive impairment and post-stroke non-cognitive impairment. The results of the analysis showed that NLR levels were significantly higher in the PSCI group than in the non-PSCI group (WMD: 1.12; 95% CI: 0.85, 1.40; *p* < 0.00001) (Figure 2A), with statistically significant heterogeneity (*I*^2^ = 80%, *p* < 0.00001).

#### 3.2.2 Correlation between PLR levels and PSCI

Six studies compared PLR levels between patients with post-stroke cognitive impairment and patients with post-stroke non-cognitive impairment, revealing significant heterogeneity among the studies (*I*^2^ = 97%, *p* < 0.00001). A meta-analysis based on a random-effects model showed that PLR levels were significantly higher in the PSCI group than in the non-PSCI group (WMD: 16.80; 95% CI: 4.30, 29.29; *p* = 0.008) (Figure 2B).

#### 3.2.3 Correlation between HALP scores and PSCI

Two studies compared HALP scores in patients with post-stroke cognitive impairment with those in patients with post-stroke non-cognitive impairment. Pooled analysis showed no statistically significant difference in HALP scores between the PSCI group and the non-PSCI group (WMD: −12.78; 95% CI: −25.95, 0.38; *p* = 0.06) (Figure 2C), with significant heterogeneity (*I*^2^ = 96%, *p* < 0.00001).

#### 3.2.4 Correlation between lymphocyte count and PSCI

Six studies compared lymphocyte count in patients with post-stroke cognitive impairment and those with post-stroke non-cognitive impairment. Pooled analysis showed no statistically significant difference in lymphocyte count between the PSCI group and the non-PSCI group (WMD: -0.13; 95% CI: −0.34, 0.07; *p* = 0.20) (Figure 2D), with significant heterogeneity (*I*^2^ = 77%, *p* = 0.0005).

### 3.3 Subgroup analysis

Subgroup analysis based on study design and country (Table 3). In case–control, prospective, and retrospective cohort studies conducted in China or Korea, increased PLR levels in patients with PSCI were statistically significant. In case–control studies, the subgroup results of the association between increased PLR levels and PSCI were not significant. However, in prospective cohort studies, PSCI was associated with a statistically significant decrease in lymphocyte count.

**Table 3:** Subgroup analysis for association between NLR, PLR, and lymphocyte count with PSCI.

Subgroup	NLR				PLR				Lymphocyte count			
	Study	WMD [95%CI]	*p* value	*I* ^2^	Study	WMD [95%CI]	*p* value	*I* ^2^	Study	WMD [95%CI]	*p* value	*I* ^2^
Total	11	1.12 [0.85, 1.40]	<0.00001	80%	6	16.80 [4.30, 29.2]	0.008	97%	6	-0.13 [−0.34,0.07]	0.0005	77%
Study design												
Prospective cohort	5	1.36 [0.82, 1.91]	<0.00001	90%	1	21.33 [14.87, 27.78]	<0.00001	NA	2	−0.30 [−0.52, −0.08]	0.007	75%
Retrospective cohort	1	1.20 [0.47, 1.93]	0.001	NA	1	11.31 [3.76, 18.87]	0.003	NA				
Case–control	5	0.95 [0.64, 1.26]	<0.00001	57%	4	17.11 [−0.02, 34.23]	<0.00001	98%	4	−0.01 [−0.25, 0.24]	0.96	56%
Study country												
China	10	1.12 [0.83, 1.42]	<0.00001	82%	6	16.80 [4.30, 29.2]	0.008	97%	6	−0.13 [−0.34,0.07]	0.0005	77%
Other	1	1.20 [0.47, 1.93]	0.001	NA								

NLR, neutrophil-to-lymphocyte ratio; PLR, platelet-to-lymphocyte ratio; NA, not applicable; WMD, weighted mean difference; CI, confidence interval.

### 3.4 Sensitivity analysis

**Figure 3:**
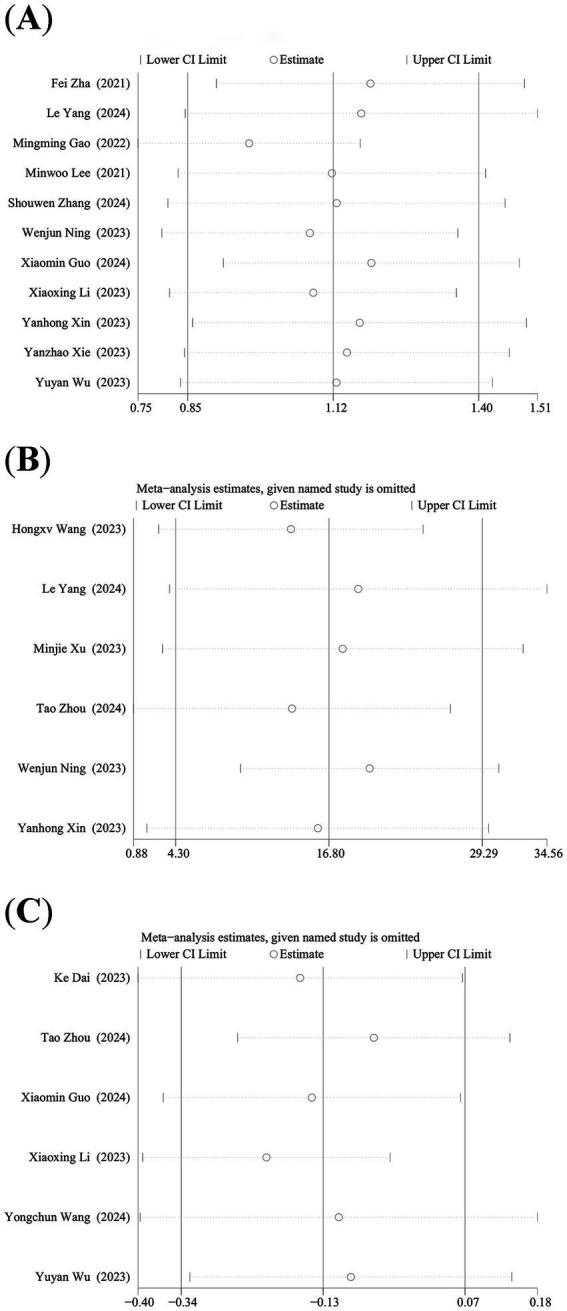
Sensitivity analysis of **(A)** NLR, **(B)** PLR, and **(C)** lymphocyte count.

We performed the leave-one-out analysis of the results of NLR levels, PLR levels, and lymphocyte count to assess the effect of each study on combined WMD. Sensitivity analysis showed that NLR levels (Figure 3A) and PLR levels (Figure 3B), the combined WMD remained unchanged after the leave-one-out analysis. However, in the sensitivity analysis of lymphocyte count (Figure 3C), the combined WMD changed after excluding the data reported by Li et al. (28).

### 3.5 Publication bias

**Figure 4:**
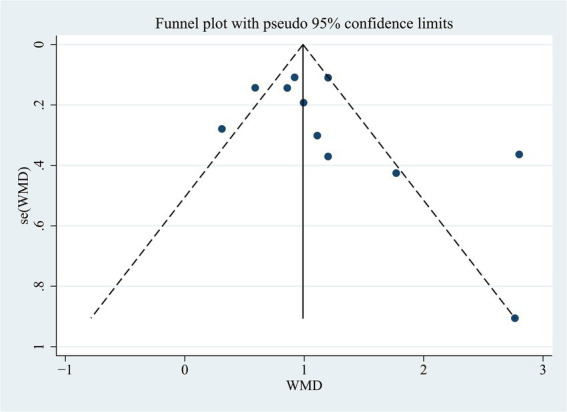
Funnel plots of NLR.

We examined whether there was a publication bias in the results of the correlation between NLR levels and PSCI. The results of Egger’s test (*p* = 0.194) or the funnel plot indicated that there was no publication bias (Figure 4).

## 4 Discussion

The primary objective of this meta-analysis was to assess the predictive value of the lymphocyte-related inflammation index for PSCI. A total of 16 articles involving 3,406 patients were included in our analysis. The results of the meta-analysis indicated that PLR and NLR levels were significantly higher in the PSCI group than in the non-PSCI group, whereas HALP scores and lymphocyte count in the PSCI group were not significantly different from the non-PSCI group.

At present, a great deal of research suggests a strong link between cognitive impairment or dementia and inflammatory factors (35, 36). Klulesh et al. revealed that patients with executive cognitive impairment had significantly higher cerebrospinal fluid concentrations of (Interleukin) IL-1β, IL-10, and serum IL-6 levels than cognitively normal patients (37). Qi et al. discovered a strong link between inflammatory factors (Tumor necrosis factor-*α*, C-reactive protein, and IL-6) and the development of vascular dementia (38). However, these inflammatory factors require additional testing to obtain, which greatly limits their use as an effective means of early screening and detection of PSCI. In recent years, an increasing number of studies have focused on inflammatory indices associated with lymphocytes, such as PLR and NLR. These two novel inflammatory indices reflect the balance between lymphocyte and platelet and neutrophil levels, respectively, and have the advantage of being more readily available and more widely used in clinical practice. In previous studies, patients with post-stroke cognitive impairment had higher levels of PLR and NLR compared to patients with post-stroke non-cognitive impairment (26, 33), which is consistent with our findings.

When a stroke occurs, numerous platelets and neutrophils accumulate in the damaged area of the brain. Platelets secrete pro-inflammatory factors into the damaged area, further exacerbating the inflammatory response and causing damage to blood vessels and neurons (39, 40), while neutrophils also induce pro-inflammatory factors, including IL-6 and matrix metalloproteinase-9, which can disrupt the brain’s blood-oxygen barrier, impeding the flow of oxygen and nutrients to the brain (41–43). Both the above-mentioned platelet and neutrophil damage to the central nervous system after a stroke cause cognitive decline, contributing to the development of PSCI. Lymphocytes, by contrast, play a role in tissue repair and neuroprotection. It has been shown that during a stroke, regulatory T lymphocytes play a neuroprotective role by producing anti-inflammatory factors and thus inhibiting the inflammatory process. However, lymphocytes undergo a corticosteroid response, resulting in their decreased numbers, which hampers the repair of post-stroke damage and accelerates the development of post-stroke cognitive impairment (44, 45). Considering the findings from previous studies indicating that PLR and NLR are strongly associated with prognosis, mortality, and severe atherosclerosis in post-stroke patients (42, 46, 47). Therefore, we suggest that PLR and NLR levels can be used as a new and effective tool for predicting PSCI, which can help in early clinical detection and prediction of PSCI.

The HALP scores is another lymphocyte-related indicator that reflects not only the patient’s inflammatory state but also their nutritional status. It is derived from the weighted sum of four items: hemoglobin, albumin, lymphocytes, and platelets, which excludes the interference of other factors, with the advantages of being easy to obtain, simple, and effective (24). The albumin and hemoglobin included in the HALP scores are important indicators of the nutritional status of the body. Studies have shown that reduced hemoglobin leads to decreased oxygen transport capacity and brain oxygen delivery, which can lead to neuronal damage, mitochondrial disorders, oxidative stress, and inflammation (48, 49). Secondly, reduced albumin levels impair the body’s ability to fight oxidation and capture oxygen-free radicals. All these factors increase the risk of poor prognosis and cognitive impairment in post-stroke patients (50). Zuo et al. (51)study results showed that low HALP scores increase the risk of PSCI. However, in our study, the HALP scores of patients in the PSCI group were not significantly different from those of patients in the non-PSCI group. We believe that there may be the following reasons for this difference: first, the number of studies included was small; second, the cognitive assessment scales used in the studies we included were not the same, which may have led to differences in the diagnosis of cognitive impairment in the patients, thus affected the final pooled results. In conclusion, we believe that the HALP scores may be able to be used as another lymphocyte-associated inflammation index to reflect the body’s inflammatory and nutritional status for early screening of PSCI, but studies with larger sample sizes are needed to validate this conclusion.

Finally, our study showed no significant difference in lymphocyte count between the PSCI and non-PSCI groups. In the results of sensitivity analysis, when the lymphocyte count data of Li et al. were excluded, there was a change in the combined WMD. The reason for this may be that unlike other studies where patients were assessed for cognitive function at a short period of time, the time point for PSCI assessment in the study by Li et al. was the 6th month after stroke, which may have led to an alteration in the combined WMD. Meanwhile, Wang et al. (25) mentioned in their study that lymphocyte count could not be identified as an independent risk factor for PSCI. Based on these findings, lymphocyte count cannot yet be considered strongly correlated with the occurrence of PSCI. Thus, it is not recommended to rely solely on lymphocyte count as an indicator for predicting the occurrence of PSCI.

Our study also has several limitations. Firstly, most of the studies we included were from China, thus limiting the generalization of the findings to other regions and ethnicities. Secondly, we used only published articles in English and Chinese, which may have led to selection bias in our findings. Also, despite our sensitivity and subgroup analysis, we still did not find a source of high heterogeneity in our study results, taking into account potential confounders, which reduces the reliability of our study results. Finally, PLR, NLR, and HALP scores in the included studies were measured only once, failing to capture dynamic changes associated with PSCI. Therefore, future studies should involve continuous monitoring to establish the association between dynamic changes in these indices and PSCI. Certainly, our study is the first meta-analysis about the predictive value of lymphocyte-associated inflammation index in PSCI, providing evidence-based medical support for early screening and prediction of PSCI in clinical practice.

## 5 Conclusion

In conclusion, increased levels of NLR and PLR were significantly associated with PSCI. However, there was no strong evidence of a direct relationship between HALP scores, lymphocyte count, and PSCI. Considering the limitations of this paper such as regional selective bias, potential heterogeneity, and retrospective study design, more prospective studies with large sample sizes and multicenter are needed in the future to further confirm the predictive value of lymphocyte-associated inflammation index for PSCI.

## Data Availability

The original contributions presented in the study are included in the article/Supplementary material, further inquiries can be directed to the corresponding author.
