# Supplementary material for: Predictive value of lymphocyte-associated inflammation index in post-stroke cognitive impairment: a systematic review and meta-analysis
Source: Front Neurol. 2025 Jan 15;15:1469152. doi: 10.3389/fneur.2024.1469152 (PMC11778338; doi:10.3389/fneur.2024.1469152)
Supplement: Supplementary file 2 [file Table_2.DOCX]

**Table1. China Knowledge Network (CNKI) search strategy**

| #1主题：脑卒中 + 缺血性脑卒中 + 急性脑卒中 +脑梗死 + 卒中后认知障碍 + 认知障碍 + 痴呆 |
| --- |
| #2摘要：淋巴细胞 |
| #3 #1 AND #2 |

**Table2. WangFang Data search strategy**

| #1题名或主题词：脑卒中 OR 缺血性脑卒中 OR 急性脑卒中 OR 脑梗死 OR 卒中后认知 |
| --- |
| 障碍 OR 认知障碍 OR 痴呆 |
| #2题名或主题词：淋巴细胞 |
| #3 #1 AND #2 |

**Table3. Web of science search strategy**

| #1 ((((TS=(Stroke)) OR TS=(apoplexy)) OR TS=(haemorrhagic stroke)) OR TS=(cerebral infarction)) OR TS=(post-stroke cognitive dysfunction) |
| --- |
| #2 TS=(Lymphocyte) |
| #3 #1 AND #2 |

**Table4. Embase search strategy**

| #1 stroke:ab,ti #2 'Lymphocyte'/exp #3 'cerebral infarction':ab,ti #4 'cerebrovasular accident'/exp #5 'haemorrhagic stroke':ab,ti #6 'post-stroke cognitive dysfunction':ab,ti #8 lymphocyte:ab,ti |
| --- |
| #9 #1 OR #3 OR #4 OR #5 OR #6 OR #7 |
| #10 #2 OR #8 |
| #11 #9 AND#10 |
| #12 'cognition'/exp #13 cognition:ab,ti #14 'cognitive dysfunction':ab,ti |
| #15 #12 OR #13 OR #14 |
| #16 #11 AND #15 |

**Table5. Cochrane search strategy**

| #1 MeSH descriptor: [stroke] explode all trees |
| --- |
| #2 MeSH descriptor: [Lymphocytes] explode all trees |
| #3 (stroke):ti,ab,kw OR (cerebral infarction):ti,ab,kw OR (haemorrhagic stroke):ti,ab,kw OR |
| (apoplexy):ti,ab,kw OR (post-stroke cognitive dysfunction):ti,ab,kw |
| #4 (Lymphocytes):ti,ab,kw |
| #5 #1 OR #3 |
| #6 #2 OR #4 |
| #7 #5 AND #6 |

**Table6. Pubmed search strategy**

| #1 "Stroke"[Mesh] |
| --- |
| #2"Lymphocytes"[Mesh] |
| #3 stroke[Title/Abstract] #4 cerebral infarction[Title/Abstract] #5haemorrhagic stroke[Title/Abstract] #6 apoplexy[Title/Abstract] #7post-stroke cognitive dysfunction[Title/Abstract] #8 Lymphocyte[Title/Abstract] |
| #9 #1 OR #3 OR #4 OR #5 OR #6 OR #7 |
| #10 #2 OR #8 |
| #11 #9 AND #10 |
